# Biotransformation of zearalenone to non-estrogenic compounds with two novel recombinant lactonases from *Gliocladium*

**DOI:** 10.1186/s12866-024-03226-3

**Published:** 2024-03-07

**Authors:** Zongping Sun, Yuting Fang, Yaohuan Zhu, Wen Tian, Junjie Yu, Jun Tang

**Affiliations:** 1 Anhui Province Key Laboratory of Environmental Hormone and Reproduction, Anhui Province Key Laboratory of Embryo Development and Reproductive Regulation, Fuyang Normal University, Anhui Fuyang, 236037 China; 2 The Anhui Agricultural University’s Comprehensive Experimental Station in the Northwest of Anhui Province, Linquan Modern Agricultural Technology Cooperation and Extension Service Center, Anhui Linquan, 236400 China

**Keywords:** Lactonases, *Gliocladium*, Zearalenone detoxification, ZEA-degrading enzyme, Mycotoxins

## Abstract

**Background:**

The mycotoxin zearalenone (ZEA) produced by toxigenic fungi is widely present in cereals and its downstream products. The danger of ZEA linked to various human health issues has attracted increasing attention. Thus, powerful ZEA-degrading or detoxifying strategies are urgently needed. Biology-based detoxification methods are specific, efficient, and environmentally friendly and do not lead to negative effects during cereal decontamination. Among these, ZEA detoxification using degrading enzymes was documented to be a promising strategy in broad research. Here, two efficient ZEA-degrading lactonases from the genus *Gliocladium*, ZHDR52 and ZHDP83, were identified for the first time. This work studied the degradation capacity and properties of ZEA using purified recombinant ZHDR52 and ZHDP83.

**Results:**

According to the ZEA degradation study, transformed *Escherichia coli* BL21(DE3) PLySs cells harboring the *zhdr52* or *zhdp83* gene could transform 20 µg/mL ZEA within 2 h and degrade > 90% of ZEA toxic derivatives, α/β-zearalanol and α/β-zearalenol, within 6 h. Biochemical analysis demonstrated that the optimal pH was 9.0 for ZHDR52 and ZHDP83, and the optimum temperature was 45 °C. The purified recombinant ZHDR52 and ZHDP83 retained > 90% activity over a wide range of pH values and temperatures (pH 7.0–10.0 and 35–50 °C). In addition, the specific activities of purified ZHDR52 and ZHDP83 against ZEA were 196.11 and 229.64 U/mg, respectively. The results of these two novel lactonases suggested that, compared with ZHD101, these two novel lactonases transformed ZEA into different products. The slight position variations in E126 and H242 in ZDHR52/ZEA and ZHDP83/ZEA obtained via structural modelling may explain the difference in degradation products. Moreover, the MCF-7 cell proliferation assay indicated that the products of ZEA degradation using ZHDR52 and ZHDP83 did not exhibit estrogenic activity.

**Conclusions:**

ZHDR52 and ZHDP83 are alkali ZEA-degrading enzymes that can efficiently and irreversibly degrade ZEA into non-estrogenic products, indicating that they are potential candidates for commercial application. This study identified two excellent lactonases for industrial ZEA detoxification.

**Supplementary Information:**

The online version contains supplementary material available at 10.1186/s12866-024-03226-3.

## Background

Mycotoxins are notorious secondary metabolites of certain fungi that potentially exist at various time points in crops and their products [1]. Since mycotoxins are relatively stable during the usual processing steps, primary exposure occurs mainly through ingestion of contaminated crops [2]. Notably, zearalenone (ZEA) is a nonsteroidal estrogenic mycotoxin mainly produced by *Fusarium* species that infects cereals in cool and humid seasons [3]. The biotransformation of ZEA into α-/β-zearalanol (ZAL) or α-/β-zearalenol (ZEL) is one of the main in vivo and in vitro metabolic processes, respectively [4, 5]. ZEA and some of its metabolites display estrogenic activities since they can occupy the same receptor region as 17β-estradiol, particularly the more estrogenically active α-zearalanol [6]. Consequently, ZEA frequently leads to fertility disorders in livestock, leading to severe economic loss in the breeding industry. Moreover, ZEA was shown to be related to carcinogenicity, genotoxicity, and immunotoxicity in living beings [7]. Due to the prevalence of ZEA contamination in Europe, Asia, America, and other regions, the danger of ZEA has attracted increasing amounts of attention. ZEA has become a regulated mycotoxin in many countries, and the detoxification of ZEA has been an urgent area of research [7].

Strategies explored to control ZEA contamination include physical, chemical, and biological methods. The former two are widely used and have been developed to mitigate ZEA in feed, while the latter is more attractive due to its high specificity, biosafety, and cost efficiency [1]. In the 1980s, several important papers documented products with far less estrogenic activity than ZEA after the transformation of ZEA by some fungi and actinomycetes [8, 9]. In particular, *G. roseum* NRRL 1859 could cleave the lactone bond of ZEA in high yield, and the product exhibited far less estrogenic activity than ZEA [10]. Kakeya et al. screened another strain, *Clonostachys rosea* (synonym: *G. roseum*) IFO 7063, which mainly transformed ZEA into a dihydroxyphenyl derivative with an open side chain, and the product did not show potential estrogenic activity in the MCF-7 cell proliferation assay [11]. The lactonase (named ZHD101) responsible for ZEA detoxification was further purified, and its gene (*zhd101*) was cloned and characterized [12]. The gene *zhd101* was successfully expressed in *Escherichia coli*, *Saccharomyces cerevisiae*, *Pichia pastoris*, and *Lactobacillus reuteri*, and the recombinant strains were able to metabolize ZEA and its isomers specifically and efficiently [13–16]. Therefore, ZEA detoxification by enzymes from microbes has received much attention.

Three typical kinds of ZEA-degrading enzymes, namely, laccases [17], peroxidases [18–20], and lactonases [16, 21–25], were identified previously. Among these enzymes, lactonase has been studied for its excellent ability to detoxify ZEA. The degradation mechanism of ZEA using lactonases ZHD101 or RmZHD was based on the cleavage of the lactone bond at C12’, which was attached by the nucleophile (Ser102) [26–28]. Moreover, the other ZEA-degrading enzyme encoded by the yeast *Trichosporon mycotoxinivorans* was predicted to cleave the macrocyclic ring of ZEA at C6’, but the crystallographic structure has not yet been solved and elucidated [29, 30]. In combination with genetic modification, maize and rice were transformed with the *gfzhd101* gene, and the resulting transgenic grains exhibited ZEA degradation, suggesting that the transgene-mediated ZEA detoxification strategy may be a promising strategy [31, 32].

To facilitate the commercial application of ZEA detoxification enzymes, researchers have adopted recombinant lactonases for industrial applications on a laboratory scale [16, 20, 24] and at the level of intact animals [5]. However, the industrial application of lactonase is limited by the properties of the enzymes, such as specific activity, thermostability, substrate specificity, and relative activity at various pH values and under other conditions. Thus, it is necessary to identify new ZEA-degrading enzymes with application-oriented enzymatic properties. In the present study, two novel ZEA-degrading lactonases, ZHDR52 and ZHDP83, from *Gliocladium* spp. were identified, and their ZEA detoxification and enzymatic properties were studied. The recombinant lactonases expressed in engineered *E. coli* cells can efficiently degrade both ZEA and its zearalenol derivatives. Moreover, the purified enzymes could efficiently degrade ZEA to non-estrogenic compounds. Notably, the two novel lactonases exhibited very high activity from 25 to 55 °C and from pH 7 to pH 10, suggesting excellent temperature and alkali tolerance. We propose two new lactonases that are suitable for the efficient and safe detoxification of ZEA in the food/feed industry under various conditions.

## Methods

### Sequence analysis, cloning, site-directed mutation, and transformation

The *zhd101* (AB076037) gene sequence for zearalenone hydrolase in *C. rosea* IFO 7063 was retrieved from the NCBI [12]. The homologous genes of *zhd101* in *G. roseum* (CGMCC No. 3.3657), *G. catenulatum* (CGMCC No. 3.3655), *G. penicilloides* (CGMCC No. 3.4252), *G. deliguescens* (CGMCC No. 3.3987), and *G. viride* (ACCC 31917) were analysed using whole-genome sequence and local BLAST. Phylogenetic analysis and multiple sequence alignment were further performed with MEGA 7.0.21 and Snapgene 4.3.6, respectively. The codons of the native genes *zhdr33*, *zhdr52*, *zhdp99*, and *zhdp83* were optimized and synthesized based on the codon bias of *E. coli* (GenScript, China).

According to multiple sequence alignment analysis and structural analysis of ZHD101 [23], the ZHDR33M and ZHDP99M mutants were constructed with site-directed mutations, including Y33L, M135L, S153V, and K158V. The synthesized genes were ligated into the pET-32a(+) vector for protein expression, and the sequence-verified plasmids were subsequently transformed into *E. coli* BL21(DE3) PLySs. The *zhd101* gene was also optimized, synthesized, cloned, and used as a positive control.

### Strains, cells, growth conditions, and chemicals

*E. coli* (ATCC 25922) and engineered *E. coli* BL21(DE3) PLySs harboring pET-32a-*zhd101*, pET-32a-*zhdr33*, pET-32a-*zhdr33m*, pET-32a-*zhdr52*, pET-32a-*zhdp99*, pET-32a-*zhdp99m*, and pET-32a-*zhdp83* were preserved in our laboratory. All the *E. coli* strains were cultured in lysogeny broth (LB) at 37 °C and 180 rpm. Ampicillin was supplemented to the LB medium at a concentration of 100 µg/ml. The medium was supplemented with 10 or 20 µg/ml ZEA, or 20 µg/ml ZEA derivatives where necessary. MCF-7 human breast cancer cells (ER+) were purchased from Hunan Fenghui Biotechnology Co., Ltd. (Changsha, China). The cells were cultured at 37 °C in an atmosphere of 5% CO_2_ in DMEM (GIBCO) supplemented with 10% fetal bovine serum (Biochannel), 100 U/mL penicillin, and 100 µg/mL streptomycin. ZEA (contents > 98%) purchased from Aladdin (Shanghai, China) and α-zearalenol (α-ZEL), β-zearalenol (β-ZEL), α-zearalanol (β-ZAL), and β-zearalanol (β-ZAL) (all contents > 98.9%) purchased from Pribolab (Qingdao, China) were dissolved in methanol as a standard stock solution (5 mg/mL). All the chemicals and reagents used were of analytical or chromatographic grade.

### ZEA degradation using engineered ***E. coli*** BL21 (DE3) PLySs

In the ZEA degradation experiment using growth cells, *E. coli* BL21(DE3) PLySs cells were cultured overnight and subsequently inoculated into fresh LB supplemented with 10 µg/mL ZEA (Aladdin, Shanghai) at a 1:100 dilution. Cultivation was performed in a shaker at 37 °C and 180 rpm. The supernatant was collected at 0 h, 2 h, 4 h, 5 h, 6 h, 7 h, 8 h, and 72 h by centrifugation and filtered through a 0.22 μm filter for further analysis. *E. coli* was used as the negative control, and all the reactions were carried out in triplicate.

In the ZEA degradation experiment using resting cells, *E. coli* BL21(DE3) PLySs cells were cultivated for 10 h and inoculated into fresh LB at a 1:100 dilution and cultured at 37 °C and 180 rpm until their turbidity reached 0.6–0.7 at 600 nm. Then, 0.1 mM isopropyl-β-D-thiogalactopyranoside (IPTG) was added, followed by overnight induction at 30 °C. Afterward, the cells were collected by centrifugation, washed with PBS (pH 7.4) twice, and resuspended in PBS (pH 7.4) containing 20 µg/mL ZEA or its derivatives to achieve an OD_600_ of 0.6. Cultivation was performed in a shaker at 37 °C and 180 rpm. The supernatant was collected at 0 h, 2 h, and 6 h by centrifugation and filtered through a 0.22 μm filter for further analysis. The cell pellets were resuspended in 1 ml of methanol and sonicated to analyse mycotoxins that may have been adsorbed. Control incubation studies were performed without cells. The degradation ability of resting cells towards ZEA and its derivatives was calculated by setting the removal rate of ZEA as 100%. The removal rate (%) was calculated as (M1–M2)/M1 × 100% (M1 is the original amount of ZEA, µg; M2 is the final amount of ZEA, µg). All the reactions were carried out in triplicate.

### Analysis of ZEA by HPLC

HPLC analyses of ZEA were carried out using an Agilent 1260 Infinity II system (Agilent, USA) equipped with a Poroshell 120 EC-C18 column (4.6 × 150 mm, 4 μm). For the detection of ZEA, samples were analysed using gradient elution with eluent A (water/0.1% formic acid/5 mM ammonium formate) and eluent B (95% methanol/5% water/0.1% formic acid/5 mM ammonium formate). The elution started at 10% B for 2 min, followed by gradient elution from 10 to 40% B over 2 min and 40 to 100% B over 8 min, followed by isocratic elution using 100% B for 5 min before decreasing to 10% B over 1 min and re-equilibration at 10% B for 1 min. The injection volume was 10 µL, and the flow rate was 0.7 mL/min. The column was kept at 30 °C, and the results were monitored by fluorescence detection at an excitation wavelength of 274 nm and an emission wavelength of 440 nm. The standard curve method was used to calculate ZEA concentration as previously described with modifications [21]. Specifically, ZEA concentrations of 0.1, 0.5, 1, 2, 5, 8, and 10 µg/mL were prepared for the analysis. Samples were eluted with 40% acetonitrile at 0.7 mL/min to detect ZEA derivatives, and the absorbance was observed at 250 nm (UV detector).

### Protein expression and purification

*E. coli* BL21(DE3) PLySs transformants were cultured for more than 10 h at 37 °C, after which 1% of the culture was inoculated into fresh LB. The cultures were grown at 37 °C to a cell turbidity of 0.6–0.7 at 600 nm and then induced overnight at 30 °C by adding 0.1 mM IPTG. The 6×His-tagged proteins were extracted by sonication in PBS (pH 7.4) containing 1 mM phenylmethanesulfonyl fluoride and 1 mM dithiothreitol, purified by a Capturem™ His-Tagged Purification Miniprep Kit (TAKARA, USA), and desalted with a Pur-A-Lyzer™ Midi Dialysis Kit (3.5 kDa) according to the manufacturer’s instructions. The concentration of proteins was analysed using a Bradford Protein Assay Kit (TAKARA, USA) with bovine serum albumin as the standard. Tris-glycine SDS‒PAGE was used to evaluate the purity of the target proteins.

### ZEA degradation activity of ZHDR52 and ZHDP83

The ZEA degradation activity of ZHD101, ZHDR52, and ZHDP83 was analysed in a 500 µL reaction mixture containing 20 µg/mL ZEA and 10 µL of the enzyme under optimal conditions (pH 9, 45 °C) for 10 min. Then, 500 µL of methanol was added to terminate the reaction, and the residues were filtered through a 0.22 μm filter for further ZEA content determination via HPLC. The specific activity (U/mg) of the proteins was calculated as previously described [21].

### Enzymatic properties of ZHDR52 and ZHDP83

The effects of pH on the relative activities of ZHD101, ZHDR52, and ZHDP83 were assessed at 4 °C for 16 h under a series of pH values using different buffers, including Na_2_HPO_4_-citric acid (500 mM, pH 5–7), Tris-HCl (1 M, pH 8–9), and glycine-NaOH (130 mM, pH 10–11). Specifically, the ZEA degradation reactions were assayed with 10 µg/mL ZEA and 10 µL purified enzyme in a 500 µL reaction mixtures, after which 500 µL methanol was added to terminate the reaction. The residues were filtered with a 0.22 μm filter for further ZEA content determination via HPLC.

The optimal temperature for the relative activity of ZHD101, ZHDR52, and ZHDP83 was determined at 25 °C, 30 °C, 35 °C, 40 °C, 45 °C, 50 °C, and 55 °C for 10 min in Tris-HCl (pH 9.0). ZEA (20 µg/mL) was used in the ZEA degradation reactions, and the ZEA concentration was determined via HPLC.

The effects of various metal ions and EDTA-2Na on the ZEA degradation properties of purified ZHD101, ZHDR52, and ZHDP83 were tested with 10 µg/mL ZEA under optimal conditions (pH 9.0, 45 °C) for 10 min. In the ZEA degradation reactions, Cu^2+^, Ni^+^, Zn^2+^, Fe^3+^, Mg^2+^, and EDTA-2Na (5 mM) were added. The enzyme activity without supplementation was used as a control and was set as 100%.

### Analyses of ZEA degradation products by thin-layer chromatography

The ZEA degradation products were prepared in 25 mL of PBS (pH 7.4) containing 400 µg of ZEA. *E. coli* BL21(DE3)PLySs cells were resuspended in the reaction mixture to an OD_600_ of 0.6, and cultivation was performed at 37 °C and 180 rpm for 3 days. The products were extracted by adding an equal volume of ethyl acetate three times, and the organic phases were combined for solvent evaporation using a rotating evaporator. Methanol (1.5 mL) was used to dissolve the products, and 500 µL of concentrated solution was obtained. A 4 µL concentrated solution was used for TLC analysis on silica gel plates with chloroform/ethyl acetate (1:1) plus 15 drops of methanol as the developing solvent. The results were observed under ultraviolet light at 254 nm.

### Estrogenic toxicity assay of ZEA degradation products

The estrogenic effects of the ZEA degradation products were analysed by the MTT cell proliferation assay [33]. MCF-7 cells were prepared at a density of 1 × 10^5^/mL and then distributed in 96-well plates (100 µL/well). The medium in each well was replaced with fresh medium containing the ZEA degradation products after the cells were attached to the wells, after which the cells were cultured for 48 h. The optical density was ultimately measured using a microplate reader at 490 nm. Each assay was carried out in triplicate.

The ZEA degradation products were prepared by the following degradation systems: overnight cultured *E. coli* or *E. coli* BL21(DE3) PLySs cells were resuspended to an OD_600_ of 0.6 in 100 mL of PBS (pH 7.4) containing 20 µg/mL ZEA. After 3 d of degradation, the degradation mixtures were vacuum freeze-dried, dissolved in 1 mL of methanol, and concentrated to 12 mg/mL hZEA (hypothetical ZEA, hypothesizing that no ZEA degradation occurred), which was used as the ZEA degradation product. Finally, hZEA was added to the cell proliferation assay mixture at final concentrations of 20, 30, and 40 µg/mL, and cells without hZEA were included as the control (for which the cell proliferation rate was set as 100%).

### Homology modelling and molecular docking simulation study of ZHDR52 or ZHDP83 with ZEA

The enzyme structures of ZHDR52 and ZHDP83 were predicted by Phyre2 using ZHD101 (PDB: 3wzl) as the template. The optimization model was further evaluated with UCLA-DOE LAB - SAVES v6.0 and preprocessed by AutoDockTooLs-1.5.7. Moreover, the 2D configuration of ZEA was downloaded from PubChem and processed via energy minimization using ChemDraw 20. Then, the ligand binding pockets of ZHDR52 and ZHDP83 were predicted using the online tool DeepSite in PlayMolecule, and the docking pocket coordinates were determined by the ProteinsPlus server of Zentrum für Bioinformatik, Universität Hamburg. Afterward, molecular docking was performed with the 3D structures of ZHDR52, ZHDP83, and ZEA using AutoDockTooLs 1.5.7, and the obtained complexes were visually analysed using PyMOL2.5.

### Statistical analysis

All the statistical analyses were performed with GraphPad Prism version 8. All the data were averaged and are expressed as the mean ± SEM. Two-way analysis of variance was used to compare the significance of differences between more than two groups. *p* values < 0.05 were considered statistically significant.

## Results

### Homology analysis and sequence alignment of the novel lactonases

**Fig. 1:**
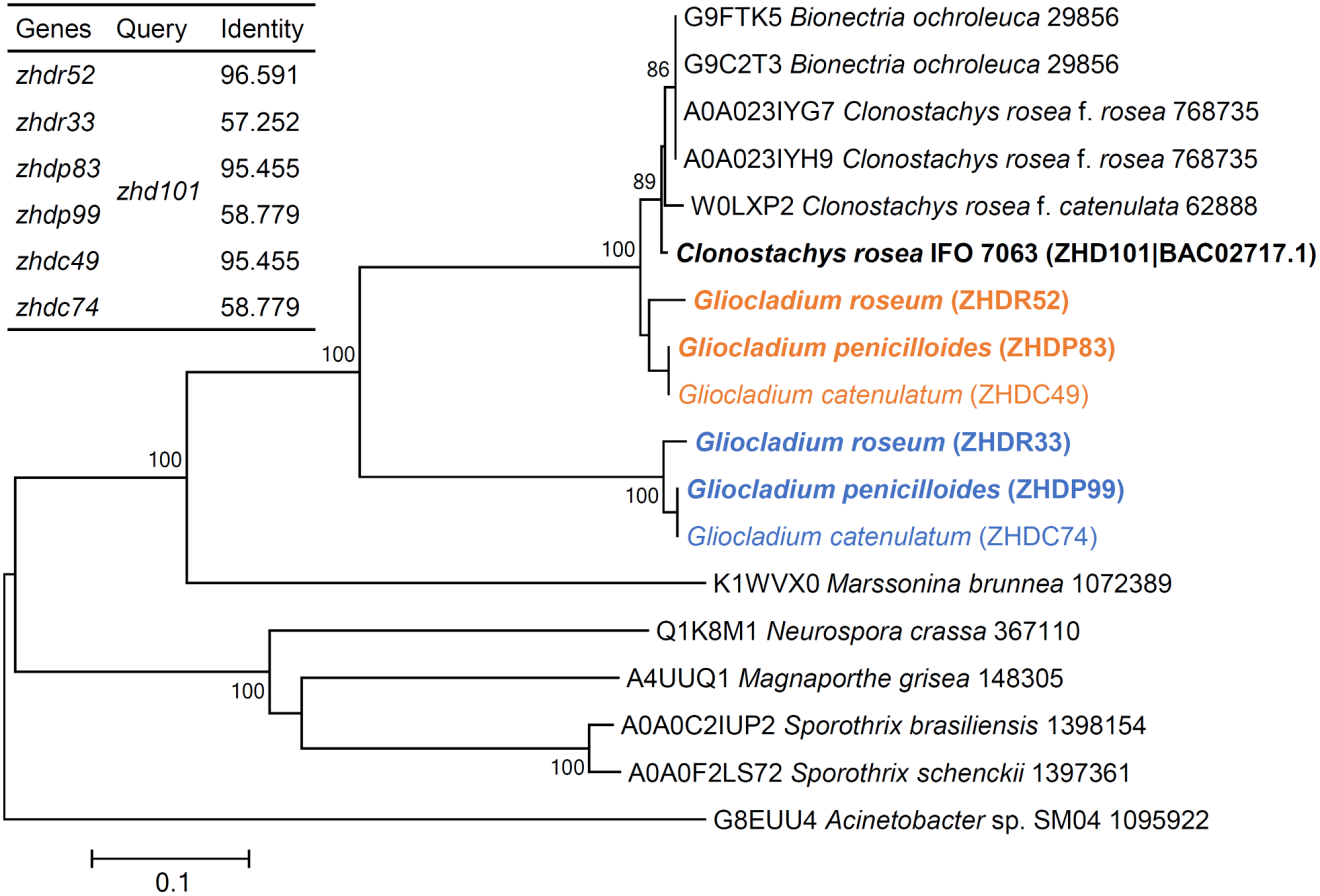
Homology analysis and phylogenetic analysis of ZEA-degrading enzymes. The tree was constructed based on the amino acid sequences using neighbor-joining analysis. The percentage of replicate trees in which the associated taxa clustered together in the bootstrap test (500 replicates) is shown next to the branches. The evolutionary distances were computed using the p-distance method

**Fig. 2:**
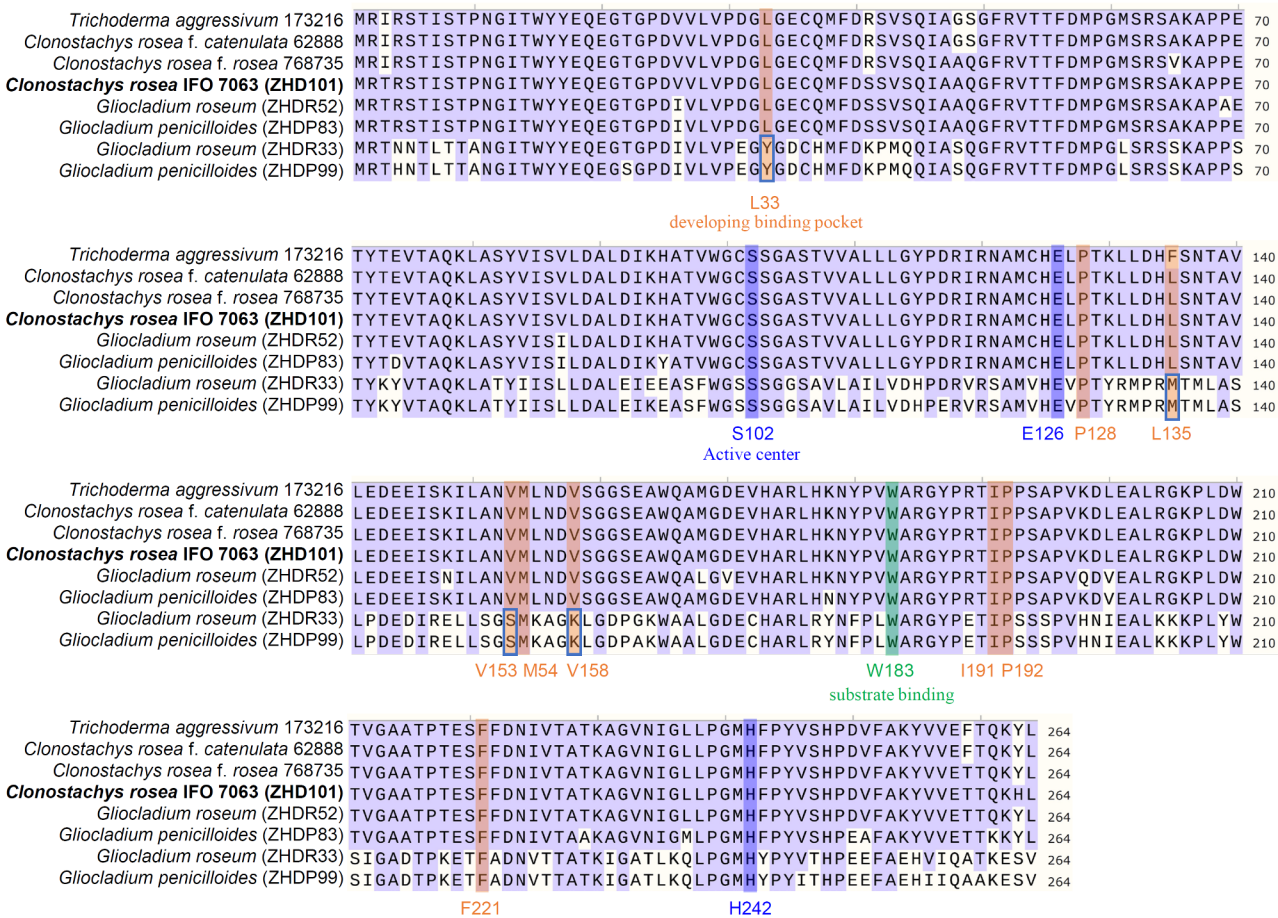
Amino acid sequence alignment of ZEA-degrading enzymes. Identical amino acids among the proteins are shown in purple, and the amino acids in boxes are selected for site-directed mutation

Six novel proteins were screened based on ZHD101 sequence information: ZHDR33 (identity 57.25%) and ZHDR52 (identity 96.59%) from *G. roseum*; ZHDP99 (identity 58.78%) and ZHDP83 (identity 95.46%) from *G. penicilloides*; and ZHDC74 (identity 58.78%) and ZHDC49 (identity 95.46%) from *G. catenulatum*. Phylogenetic analysis of the novel proteins from different microorganisms revealed close evolutionary relationships between the ZEA-degrading proteins of high identity and ZHD101 and a clear phylogenetic separation between the proteins of low identity and ZHD101 (Fig. 1). In addition, ZHDP83 and ZHDC49 clustered on the same branch and shared 100% sequence identity, similar to ZHDP99 and ZHDC74. Thus, ZHDC49 and ZHDC74 were omitted from subsequent research. Multiple sequence alignment suggested that some of the amino acid residues of ZHDR33 and ZHDP99 were different from those of other proteins, including residues that participate in the development of the binding pocket [23] (Fig. 2). After site-directed mutation, the ZHDR33M and ZHDP99M mutants and ZHD101 shared the same amino acid residues used for the development of the binding pocket.

### ZEA degradation in recombinant ***E. coli*** cells

**Fig. 3:**
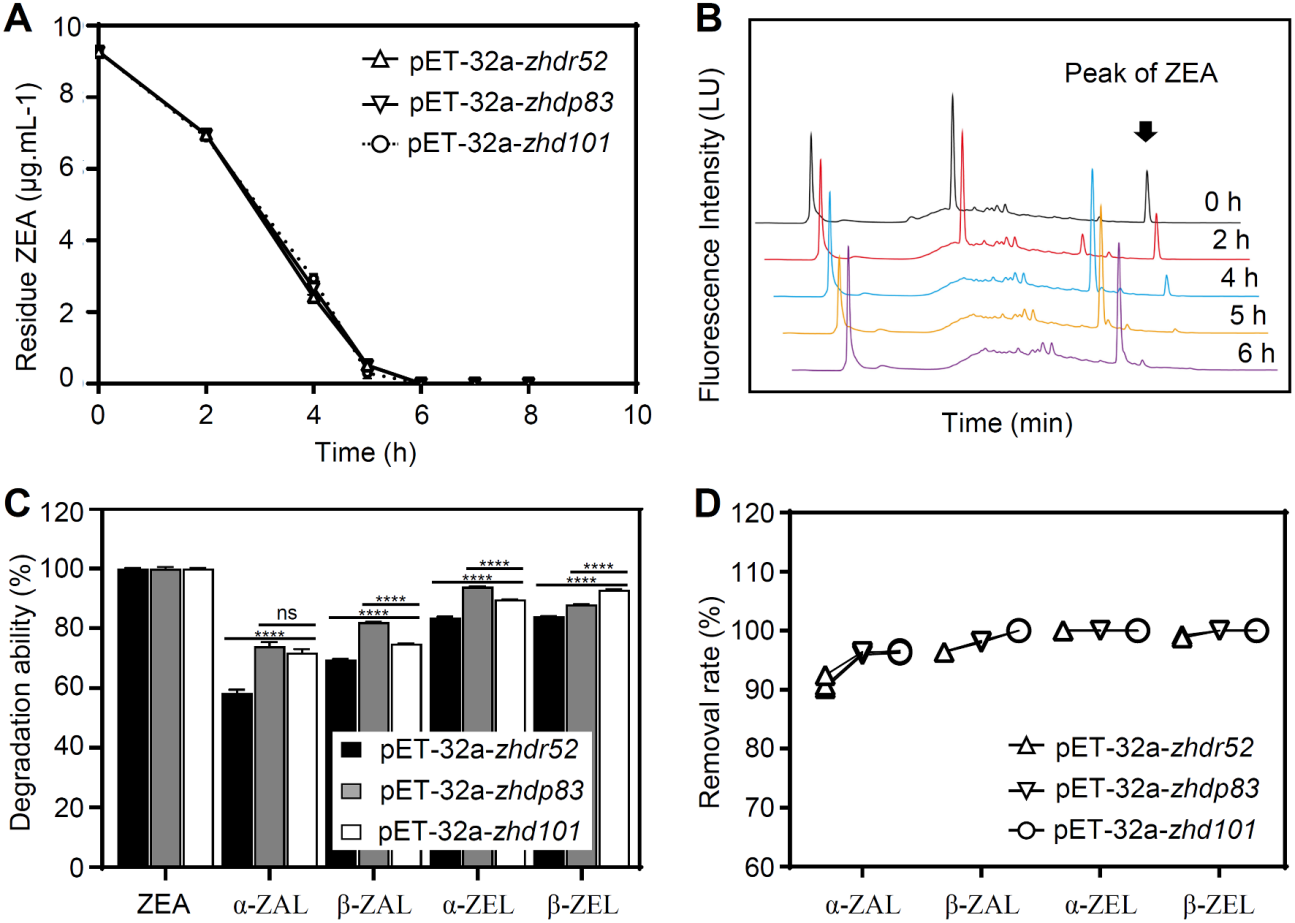
ZEA degradation in engineered *E. coli* BL21(DE3) PLySs cells. **A** and **B** represent the results of ZEA degradation in growing cells. **C** and **D** represent the degradation results of ZEA and its derivatives using resting cells induced by IPTG after 2 and 6 h, respectively. The peaks of ZEA in the representative HPLC chromatograms at each time point are indicated with black arrows. Tukey’s multiple comparisons test was used for multiple comparisons, **** *p* < 0.0001

HPLC analysis revealed that transformed *E. coli* BL21(DE3) PLySs harboring the *zhdr52* or *zhdp83* gene could degrade ZEA. However, *E. coli* BL21(DE3) PLySs transformed with the *zhdr33*, *zhdp99*, *zhdr33m*, or *zhdp99m* gene did not degrade ZEA after 3 days of incubation. Specifically, 10 µg/mL ZEA was completely degraded within 6 h by transformed *E. coli* BL21(DE3) PLySs harboring the *zhdr52*, *zhdp83*, or *zhd101* gene (Fig. 3A), which was indicated by the decreasing peaks of ZEA in the HPLC chromatograms (Fig. 3B).

After IPTG induction of *E. coli* BL21(DE3) PLySs transformants harboring pET-32a-*zhdr52*, pET-32a-*zhdp83*, and pET-32a-*zhd101*, the removal rate of ZEA (20 µg/mL) was more than 99% within 2 h. Moreover, the relative degradation ability of α-/β-ZAL or α-/β-ZEL was above 58%, and pET-32a-*zhdp83* exhibited better degradation rates of α-ZAL, β-ZAL, and α-ZEL (Fig. 3C). In addition, the removal rates of α-/β- ZAL and α-/β-ZEL (20 µg/mL) by the transformed *E. coli* BL21(DE3) PLySs were greater than 90% within 6 h, in which the removal rate of α-/β-ZEL reached 100% with pET-32a-*zhdp83* and pET-32a-*zhd101* (Fig. 3D). The results here suggested that the degradation efficiency of ZEA using the expression products of *zhdr52* or *zhdp83* was not significantly different from that of *zhd101*. The *zhdp83* gene exhibited greater degradation of α-/β-ZAL and α-ZEL than the other two genes.

### Protein expression and purification

**Fig. 4:**
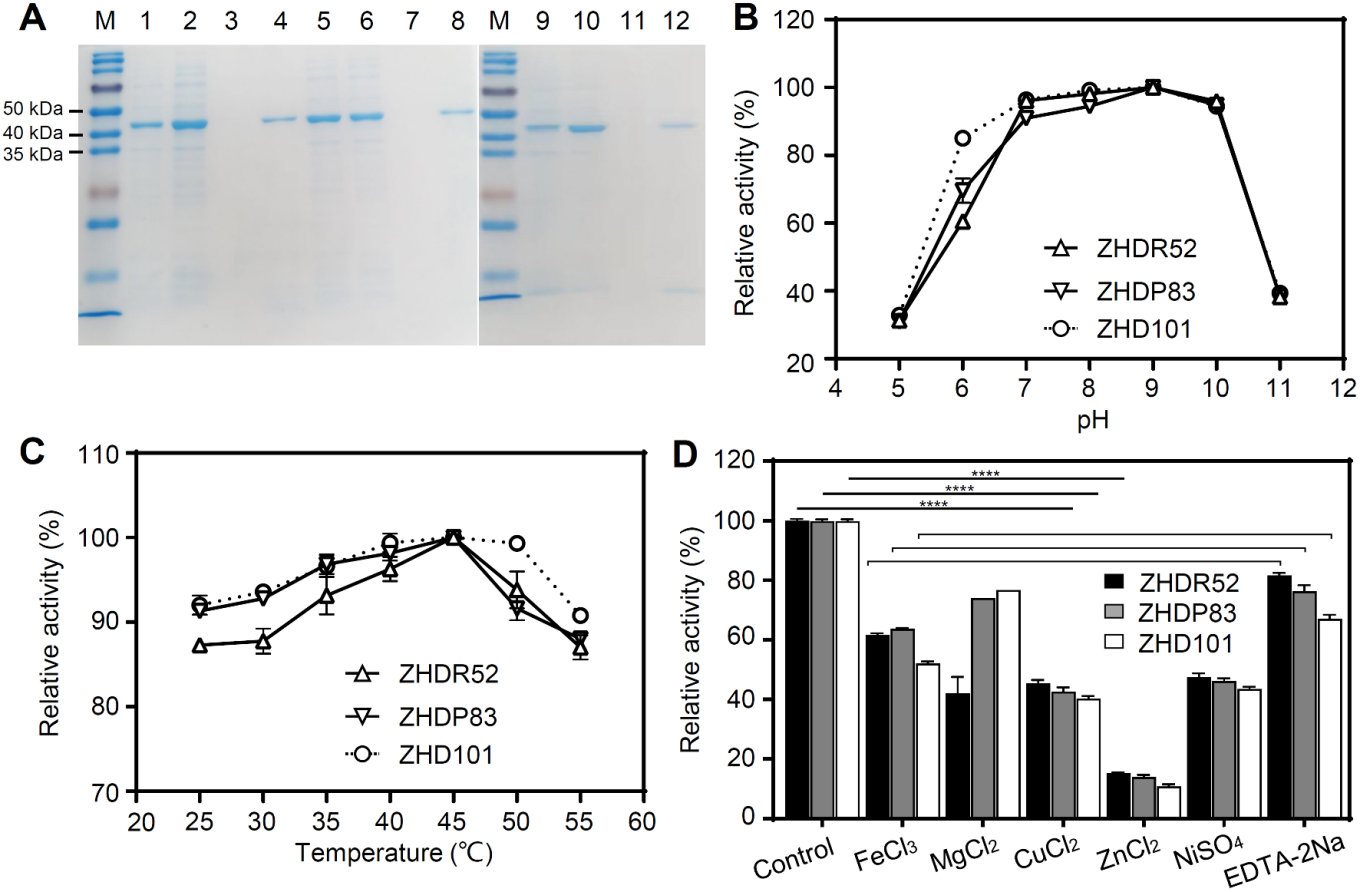
Enzymatic properties of ZEA-degrading enzymes. **A**: SDS‒PAGE analysis of the purified proteins. M: Marker; 1: crude ZHDR52; 2: 1st eluent of crude ZHDR52; 3: 2nd eluent of crude ZHDR52; 4: purified ZHDR52; 5: crude ZHDP83; 6: 1st eluent of crude ZHDP83; 7: 2nd eluent of crude ZHDP83; 8: purified ZHDP83; 9: crude ZHD101; 10: 1st eluent of crude ZHD101; 11: 2nd eluent of crude ZHD101; 12: purified ZHD101. **B**: Effect of pH on the activity of ZEA-degrading enzymes. **C**: Effects of temperature on the activity of ZEA-degrading enzymes. **D**: Relative activity of the ZEA-degrading enzymes. The data are shown as the mean ± SEM (*n* = 3). Dunnett’s multiple comparisons test was used for multiple comparisons, **** *p* < 0.0001

As shown by SDS‒PAGE analysis (Fig. 4A), the ZDHR52, ZHDP83, and ZHD101 proteins were successfully expressed by recombinant *E. coli* BL21(DE3) PLySs cells. The results of purified and desalted proteins suggested that the molecular mass of the target proteins was approximately 46.7 kDa, corresponding to the theoretical value. The purification process was evaluated by enzyme activity under optimal conditions. We found that the enzymes were stable during purification and desalting and were ultimately purified 6–7 times. Based on the enzyme activity and protein content determination, the specific activities of the purified enzymes towards ZEA were 196.11 U/mg, 229.64 U/mg, and 135.40 U/mg for ZDHR52, ZHDP83, and ZHD101, respectively (Table 1).

**Table 1:** Purification steps of ZEA-degrading proteins

Name	Purification steps	Total protein (µg)	Total activity (U)	Specific activity (U/mg)	Purification (-fold)	Yield (%)
ZHDR52	Crude	24.01	0.791	32.93	1.0	100.0
	Purified	1.47	0.288	196.11	6.0	36.4
ZHDP83	Crude	22.34	0.814	36.43	1.0	100.0
	Purified	1.24	0.284	229.64	6.3	34.9
ZHD101	Crude	26.55	0.487	18.36	1.0	100.0
	Purified	1.61	0.218	135.40	7.4	44.8

### Enzymatic properties

The optimum pH and temperature for these two novel lactonases and the effects of metal ions on them were analysed. Specifically, the impact of pH and temperature on the enzyme activity of purified ZEA-degrading proteins toward ZEA was determined at various pH and temperature values. According to the results, ZDHR52 and ZHDP83 retained > 90% of their enzyme activity at pH values ranging from 7.0 to 10.0, with an optimum occurring at approximately pH 9.0 (Fig. 4B). Therefore, ZDHR52 and ZHDP83 are alkali ZEA-degrading enzymes, similar to ZHD101. When the degradation of ZEA was performed from 25 to 55 °C, both ZDHR52 and ZHDP83 exhibited > 85% enzyme activity, indicating that these two enzymes are heat stable (Fig. 4C). The optimal temperature for effective enzyme activity of ZDHR52, ZHDP83, and ZHD101 was 45 °C. Moreover, there was no significant difference among them.

As shown in Fig. 4D, the addition of Fe^3+^, Mg^2+^, and EDTA-2Na inhibited the ZEA-degrading activity of ZDHR52, ZHDP83, and ZHD101, and more than 50% of the enzymatic activity was lost in the presence of Cu^2+^ and Ni^2+^. Furthermore, Zn^2+^ abolished nearly 90% of the enzymatic activity of these three enzymes. In addition, Mg^2+^ more significantly inhibited the activity of ZDHR52 than it did for both ZHDP83 and ZHD101.

### Analysis of the ZEA degradation products

**Fig. 5:**
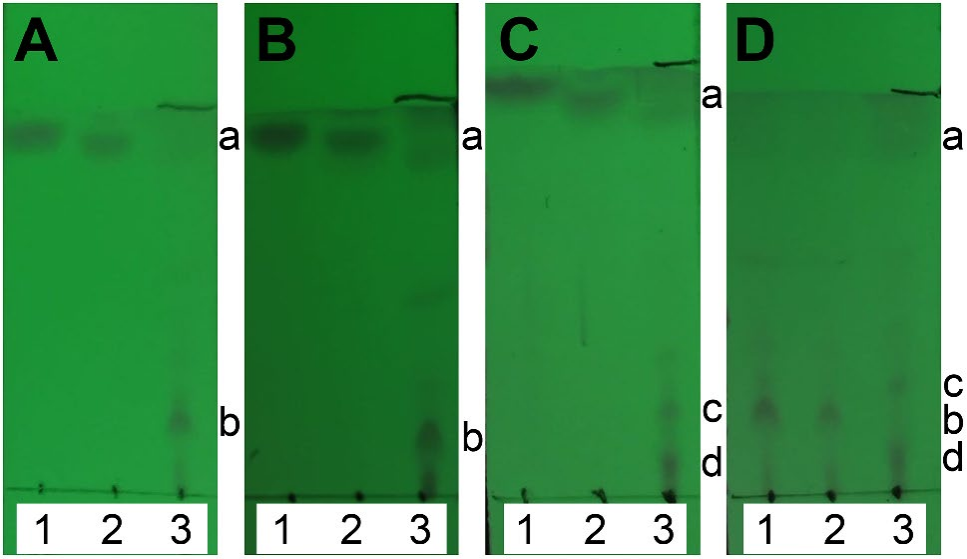
TLC analysis of the ZEA degradation products produced by transformed *E. coli* BL21 (DE3) PLySs cells. Lanes 1 and 2 in A, B, and C represent the standard ZEA and the ZEA degradation products by *E. coli* (negative control), respectively; lane 3 in A, B, and C represent the ZEA degradation products by ZHDR52 (**A**), ZHDP83 (**B**), and ZHD101 (**C**), respectively; (**D**) represents the ZEA degradation products by ZHDR52 (lane 1), ZHDP83 (lane 2), and ZHD101 (lane 3), respectively. The position of ZEA (compound a) and the main products (compounds b-d) are indicated

To compare the biotransformation products of ZEA catalyzed by ZHDR52, ZHDP83, and ZHD101 expressed as recombinant forms in *E. coli* BL21(DE3)PLySs, the resulting products were extracted and developed on a TLC plate (Fig. 5). The product of the negative control contained only ZEA (compound a), which was present at the same position as the standard ZEA. Surprisingly, the products of ZHDR52 and ZHDP83 were mainly compound b, while the products of ZHD101 consisted of a majority of compound c and a certain amount of compound d. These results indicated that the degradation products of ZEA using ZHDR52 and ZHDP83 may be the same, but they were probably different from those catalyzed by ZHD101.

**Fig. 6:**
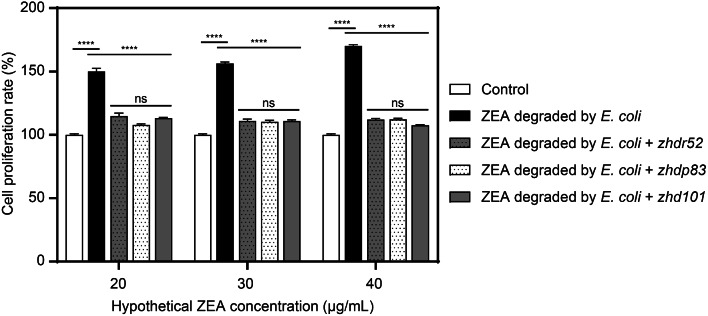
The cell proliferation rate of MCF-7 cells treated with the ZEA degradation products. Dunnett’s multiple comparisons test was used for multiple comparisons, **** *p* < 0.0001

Since ZEA and some of its metabolites stimulate the growth of human breast cancer cells, MCF-7 cells were used to estimate the estrogenic activity of ZEA degradation products (Fig. 6). Compared with those of the control, the ZEA degradation products produced by *E. coli* significantly increased the cell number, because *E. coli* cannot degrade ZEA. In contrast, the products of ZEA transformed by ZHDR52 or ZHDP83 did not exhibit the ability to stimulate cell growth when the amount of ZEA was increased, which was consistent with what was observed for the ZEA products catalyzed by ZHD101. These results indicate that ZHDR52 and ZHDP83 can transform ZEA into non-estrogenic products but not into its α-/β-derivatives, which still exhibit estrogenic activity.

### Structural modelling of ZDHR52 and ZHDP83

**Fig. 7:**
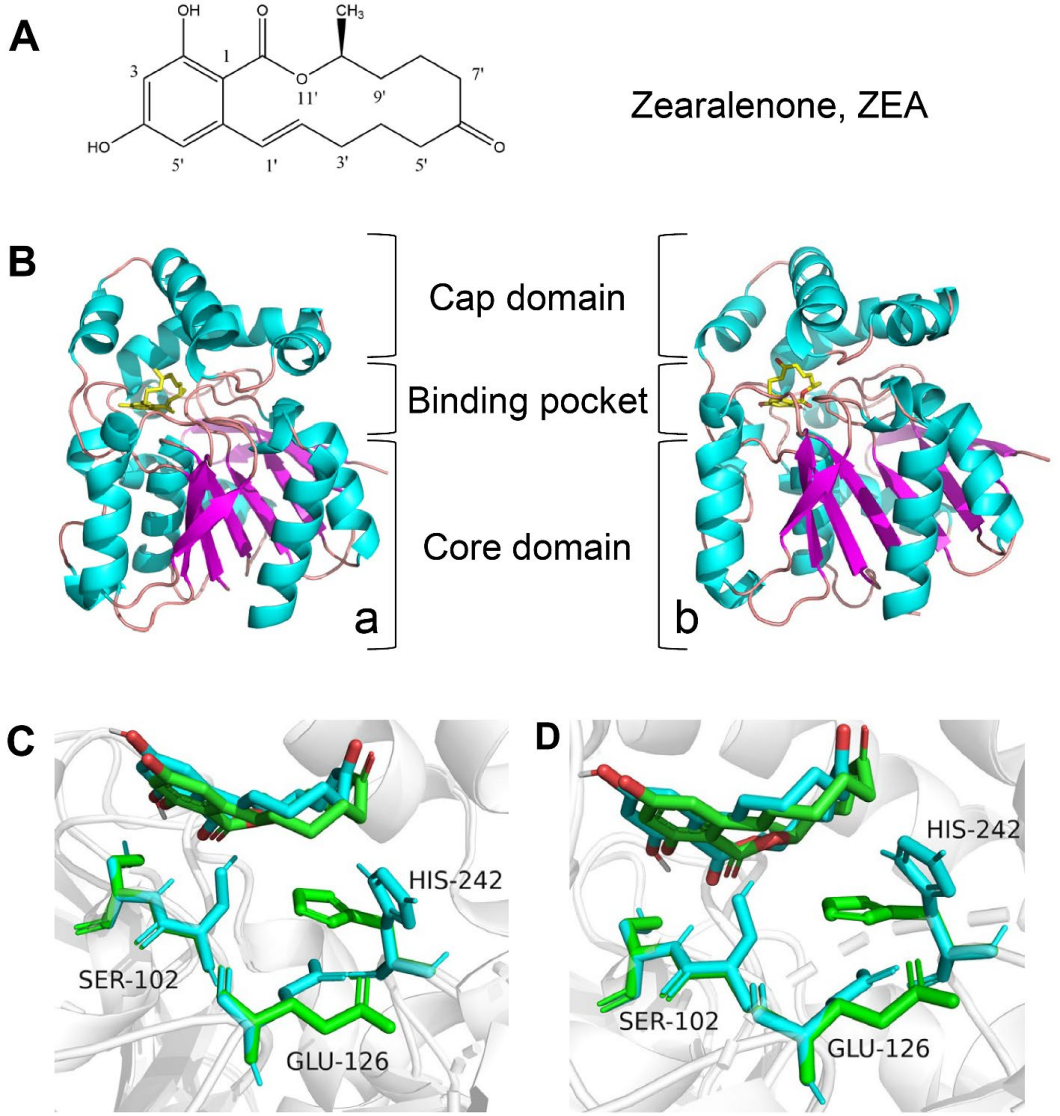
Structures of ZEA and ZEA-degrading enzymes. (**A**) Chemical structure of ZEA. (**B**) Structural panorama of ZHDR52/ZEA (**a**) and ZHDP83/ZEA (**b**). The structure of ZEA is shown as a stick model in yellow. (**C**) Catalytic triplet comparison of the superimposed structures of ZHDR52/ZEA (cyan) and ZHD101/ZEA (green). (**D**) Catalytic triplet comparison of the superimposed structures of ZHDP83/ZEA (cyan) and ZHD101/ZEA (green). The structure of ZEA is shown as stick models in cyan or green

Based on the Ramachandran plot quality assessment, the best modelling structures of ZDHR52 and ZHDP83 were highly reliably simulated (Additional file 1). The most favoured regions of ZDHR52 and ZHDP83 accounted for 92%. Moreover, there were no residues in the disallowed regions. The structures of homologous proteins of ZHD101, ZHDR52, and ZHDP83 are significantly similar. The bound ZEA from the ZHDR52/ZEA and ZHDP83/ZEA complexes was located in the deep pocket between the core and cap domains (Fig. 7A, B). Both ZHDR52 and ZHDP83 have an α-helical cap domain formed by α-helices and a core domain developed by central β-sheets and several flanking α-helices. As revealed by the superimposed structures, there is a catalytic triad of Ser102-His242-Glu126 below the substrate (Fig. 7C, D). Notably, a variation lies in the positions of E126 and H242 in ZDHR52 and ZHDP83, and the position of ZEA is slightly shifted compared with that of ZHD101/ZEA. Hydrogen bonds developed between the substrate and its surrounding amino acid residues, among which the interactions between S103, G32, W183, or Y187 and ZEA appeared to play important roles in substrate degradation (Additional file 2).

## Discussion

In recent decades, zearalenone occurrence and exposure assessment in cereals and feed materials have been performed, and the results have been analysed and documented [3, 7, 34]. Zearalenone has emerged as a severe health hazard in most feed and animal products, and climate change scenarios worldwide exacerbate this threat [2, 35, 36]. Since zearalenone significantly reduces the quality of grain products and causes serious health problems in food chains, its contamination leads to enormous economic losses and exacerbates public health issues [37]. The present situation implies an urgent need for effective detoxification methods to control ZEA pollution. Currently, the enzymatic decontamination method is regarded as a promising strategy for solving these issues due to its advantages of practicality, effectiveness, innocuity, and no secondary pollution.

ZHD101 is known to degrade ZEA into products of non-estrogenic activity in two steps: cleavage of the big ring on the lactone bond and subsequent decarboxylation [11]. The mechanism of ZEA degradation was further characterized, and the overall protein structure was determined [26]. Afterward, several homologues of ZHD101 were identified, expressed, characterized, and even modified by structure-based engineering [21, 22, 24, 27, 38]. However, the application of ZEA-degrading enzymes is still at the initial stage. New ZEA-degrading enzymes with excellent properties for ZEA detoxification during industrial processes still need to be identified. In the present research, ZHDR52 and ZHDP83, two novel lactonases sharing 95.5% and 96.6% homology with ZHD101, respectively, were successfully screened and expressed in *E. coli* BL21(DE3) PLySs. According to the results of enzymatic function analysis, these two lactonases could efficiently catalyze the degradation of ZEA into products of non-estrogenic activity instead of its α-/β-derivatives within a few hours.

The reported ZHD101 homologous proteins usually exhibit an amino acid identity of 60-98.3% to ZHD101 from *C. rosea* IFO 7063 [21, 24, 27, 38], and the amino acid residues for ZENG and ZHD101 are almost identical except for one position [22]. Although ZENC shares 29% identical residues with ZHD101, it also has the α/β hydrolase fold feature and the catalytic triad [25]. The proteins ZHDR33 and ZHDP99 from *Gliocladium* were found to have a significant level of similarity (57−58%) to ZHD101 in this study. While ZHDR33 and ZHDP99 have the same catalytic triad and substrate binding site as ZHD101, they do not exhibit the ability to degrade ZEA. Since four vital residues (nine in total) that were in direct contact with the ZEA substrate in ZHDR33 and ZHDP99 were different from those in ZHD101 [23], site-directed mutations were performed with *zhdr33* and *zhdp9*9. However, the ZHDR33m and ZHDP99m mutants were still free of ZEA degradation ability, suggesting that other vital residues or structures are essential for the degradation of the ZEA substrate by ZHD101 homologous proteins.

Notably, ZHDR52 and ZHDP83 expressed in *E. coli* BL21(DE3) PLySs exhibited excellent properties for ZEA degradation, with degradation efficiencies as high as that of ZHD101. ZEA (10 µg/mL) was completely degraded within 6 h in the transformed *E. coli* BL21(DE3) PLySs cells. When the expression of ZHDR52 and ZHDP83 was induced with IPTG, the 20 µg/mL ZEA was almost completely removed within 2 h. Moreover, a higher degradation rate of ZEA (20 µg/mL) was obtained after 10 min with purified ZHDR52 (31.4%) and ZHDP83 (33.7%) instead of ZHD101 (23.1%). Furthermore, although ZHDR52 and ZHDP83 prefer ZEA to ZALs and ZELs as substrates, they can degrade more than 90% of the ZALs and ZELs (20 µg/mL) within 6 h, and ZHDP83 exhibits increased α-/β-ZAL and α-ZEL selectivity. Since the α-derivatives of ZEA were documented to have higher estrogenic toxicity [9, 23], a better ZHDP83 activity toward toxic α-derivatives implied great potential for further industrial applications.

Given the importance of lactonase in further industrial applications, the specific activities of ZHDR52, ZHDP83, and ZHD101 were studied. According to the same method described previously [21], the enzyme activity of ZHD101 towards ZEA was 135.40 U/mg in this study, similar to the reported value (128.0 U/mg). Interestingly, the specific activities of ZHDR52 and ZHDP83 were 1.45 and 1.70 times greater than that of ZHD101, respectively, indicating that each milligram of these two novel lactonases has greater enzyme activity than that of ZHD101. Even though the optimum reaction pH and temperature for ZHDR52 and ZHDP83 were pH 9.0 and 45 °C, respectively, they maintained very high activity toward ZEA from pH 7.0 to pH 10.0 and from 25 to 55 °C, which makes these two novel lactonases better candidates for ZEA degradation applications in food and feed. Based on previous reports, Zhd518 and ZENG are two preeminent ZEA-degrading enzymes that exhibit high activity under neutral pH conditions [21, 22]. Notably, ZHDR52 and ZHDP83 retained more than 90% of their enzyme activity at pH 7.0, suggesting that these two novel lactonases exhibited excellent activity at neutral pH. Moreover, the effects of metal ions on the activity of ZHDR52 and ZHDP83 were similar to those of recombinant ZENG, Zhd518, and ZENC, which have metal-free properties [21, 22, 25].

ZHD101 was documented to cleave the big ring of ZEA at the ester bond, producing the nontoxic alkylresorcinol product with one additional hydroxy group [12, 23]. The ZEA degradation product alkylresorcinol theoretically and experimentally exhibits strong polarity [12]. According to the primary product analysis, the ZEA degradation products catalyzed by ZHDR52, ZHDP83, and ZHD101 exhibited strong polarity since the product spots on the same TLC plates were far from the solvent front, while the ZEA spots were near the solvent front. According to the HPLC analysis of ZEA degradation products, a new peak emerged and increased with decreasing ZEA peak intensity. Compared with the retention time of ZEA (15.3 min), the retention time of the new peak was approximately 13 min, indicating that the ZEA degradation product has strong polarity.

Notably, the products of ZEA degradation catalyzed by ZHDR52 and ZHDP83 were shown to be different from those of ZHD101 based on the preliminary TLC analysis. The position of the amino acid residue H242 is vital for the enzyme activity of ZEA-degrading enzymes toward ZEA and its derivatives, and variation in the H242 side chain position was reported to change the activity of ZHD518 and ZHD101 toward ZEA derivatives [21, 23]. Notably, the H242 side chain position in the catalytic triad of ZHDR52 and ZHDP83 was slightly different from that in ZHD101, which may explain the difference in ZEA degradation products. Therefore, ZHDR52 and ZHDP83 were identified as novel enzymes with excellent ZEA-degrading activities. The exact structure of the ZEA degradation products obtained using ZHDR52 and ZHDP83 will be identified in further research.

As previously stated, ZHD101 can convert ZEA into a non-estrogenic compound [11, 12]. The estrogen toxicity of the products is usually analysed using MCF-7 cells, an estrogen receptor-positive cell line [19, 39]. Thus, an MTT cell proliferation assay was performed to evaluate the estrogen toxicity of the ZEA degradation products. The proliferation rate of the MCF-7 cells verified the non-estrogenic activity of the ZEA degradation products using ZHDR52 and ZHDP83, indicating that these two novel lactonases could be used in countering ZEA contamination.

## Conclusions

To conclude, the present study screened two novel ZEA-degrading enzymes, ZHDR52 and ZHDP83, which have enormous potential for application in the food/feed industry. Based on the available data, these two recombinant lactonases could be used for irreversible ZEA detoxification under various conditions. We speculated that these two enzymes may perform ZEA detoxification via different mechanisms from those of ZHD101 and yield different non-estrogenic products. In addition, the ZEA detoxification ability of *Gliocladium* spp. strains used in this study is currently under investigation.

### Electronic supplementary material

Below is the link to the electronic supplementary material.

Supplementary Material 1 : Additional file 1: The Ramachandran plots of ZHDR52 (A) and ZHDP83 (B) were generated via template-based homology modeling.

Supplementary Material 2: Additional file 2: The superimposed structures of ZEA and the amino acid residues of ZHDR52 (A) and ZHDP83 (B). The amino acid residues (cyan or green) and ZEA (yellow) are shown as stick models. Dashed lines indicate hydrogen bonding, and the distances are indicated.

## Data Availability

No datasets were generated or analysed during the current study.

## Abbreviations

IPTG: isopropyl-β-D-thiogalactopyranoside
LB: lysogeny broth
ZAL: zearalanol
ZEA: zearalenone
ZEL: zearalenol
