# Supplementary material for: Biotransformation of zearalenone to non-estrogenic compounds with two novel recombinant lactonases from Gliocladium
Source: BMC Microbiol. 2024 Mar 7;24:75. doi: 10.1186/s12866-024-03226-3 (PMC10921726; doi:10.1186/s12866-024-03226-3)
Supplement: Supplementary file 1 — Supplementary Material 1 : Additional file 1: The Ramachandran plots of ZHDR52 (A) and ZHDP83 (B) were generated via template-based homology modeling. [file 12866_2024_3226_MOESM1_ESM.pdf]

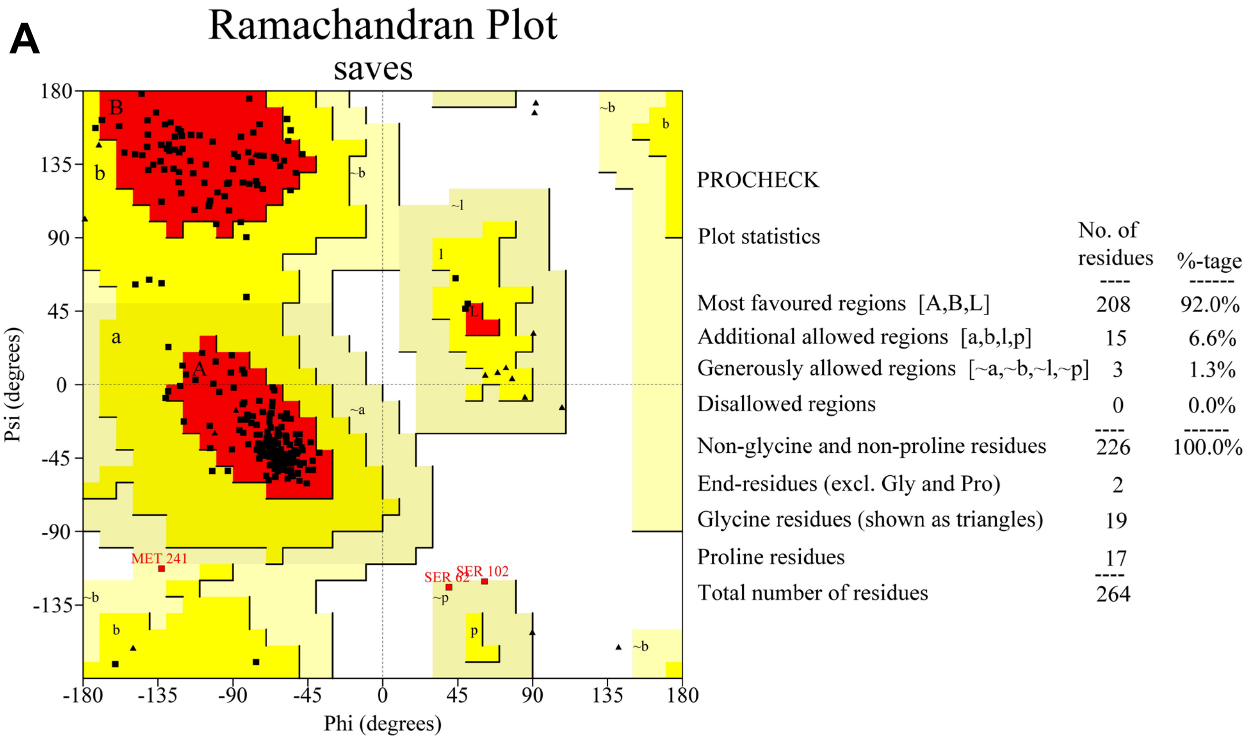

PROCHECK

Plot statistics

	No. of residues	%-tage
Most favoured regions [A,B,L]	208	92.0%
Additional allowed regions [a,b,l,p]	15	6.6%
Generously allowed regions [~a,~b,~l,~p]	3	1.3%
Disallowed regions	0	0.0%
Non-glycine and non-proline residues	226	100.0%
End-residues (excl. Gly and Pro)	2	
Glycine residues (shown as triangles)	19	
Proline residues	17	
Total number of residues	264	

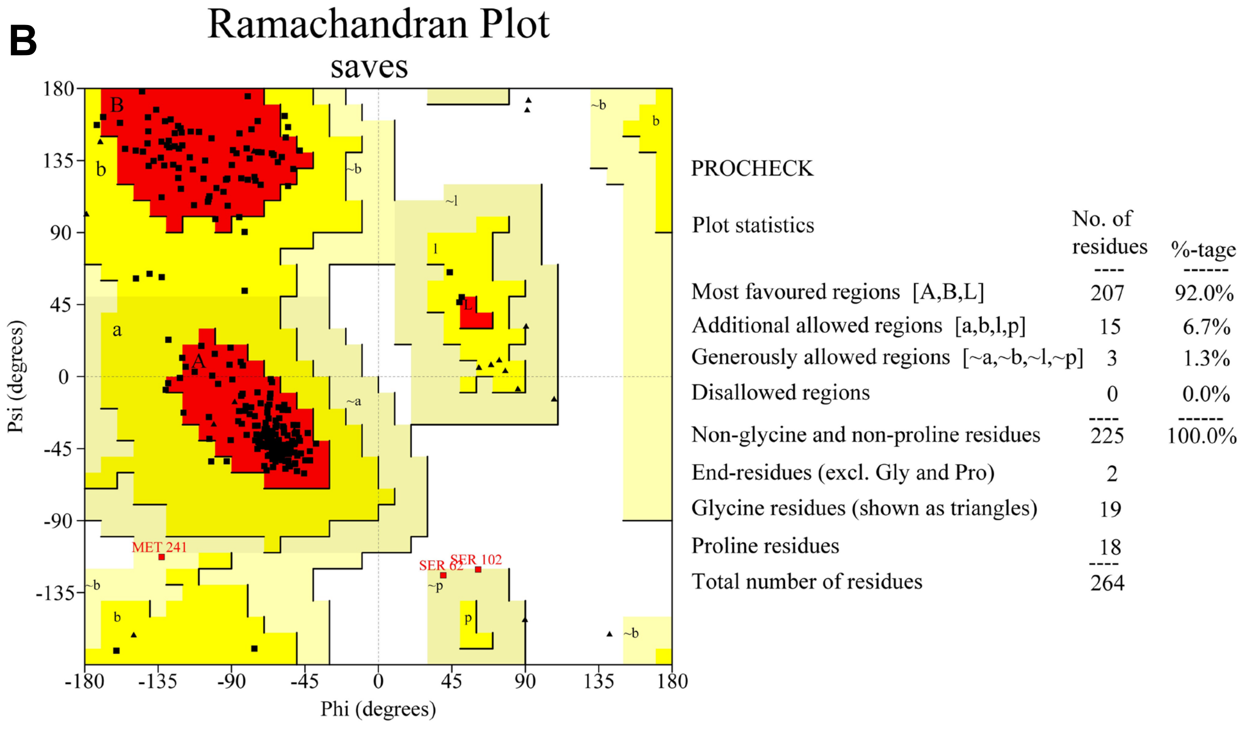

PROCHECK

Plot statistics

	No. of residues	%-tage
Most favoured regions [A,B,L]	207	92.0%
Additional allowed regions [a,b,l,p]	15	6.7%
Generously allowed regions [~a,~b,~l,~p]	3	1.3%
Disallowed regions	0	0.0%
Non-glycine and non-proline residues	225	100.0%
End-residues (excl. Gly and Pro)	2	
Glycine residues (shown as triangles)	19	
Proline residues	18	
Total number of residues	264	

Additional file 1: The Ramachandran plots of ZHDR52 (A) and ZHDP83 (B) were generated via template-based homology modeling..
