# Supplementary material for: Biotransformation of zearalenone to non-estrogenic compounds with two novel recombinant lactonases from Gliocladium
Source: BMC Microbiol. 2024 Mar 7;24:75. doi: 10.1186/s12866-024-03226-3 (PMC10921726; doi:10.1186/s12866-024-03226-3)
Supplement: Supplementary file 2 — Supplementary Material 2: Additional file 2: The superimposed structures of ZEA and the amino acid residues of ZHDR52 (A) and ZHDP83 (B). The amino acid residues (cyan or green) and ZEA (yellow) are shown as stick models. Dashed lines indicate hydrogen bonding, and the distances are indicated. [file 12866_2024_3226_MOESM2_ESM.pdf]

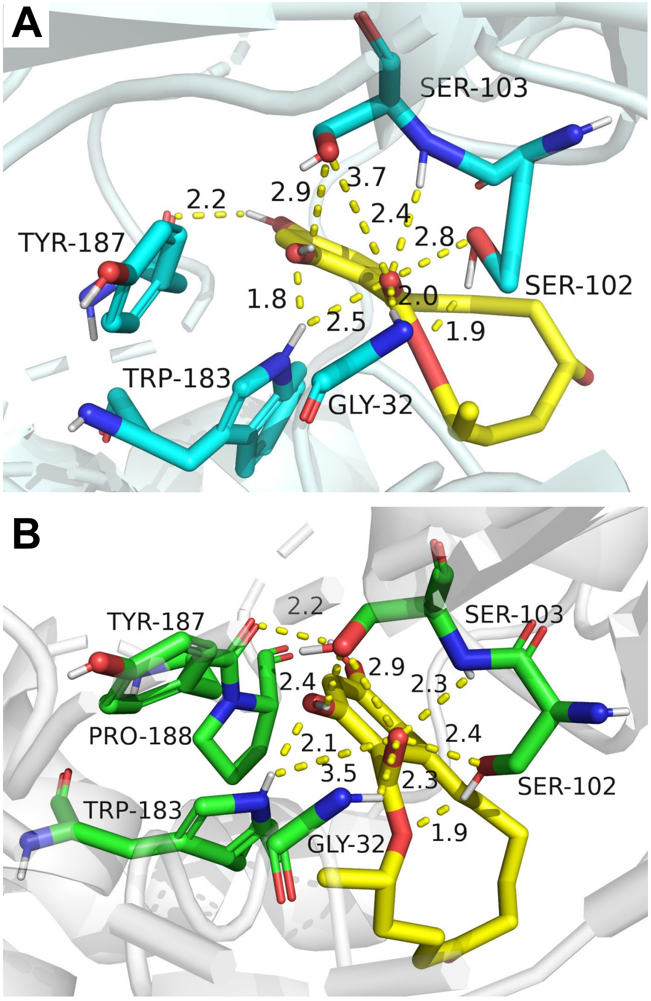

Additional file 2: The superimposed structures of ZEA and the amino acid residues of ZHDR52 (A) and ZHDP83 (B). The amino acid residues (cyan or green) and ZEA (yellow) are shown as stick models. Dashed lines indicate hydrogen bonding, and the distances are indicated.
